# Proton pump inhibitor ilaprazole suppresses cancer growth by targeting T-cell-originated protein kinase

**DOI:** 10.18632/oncotarget.16609

**Published:** 2017-03-27

**Authors:** Mengzhu Zheng, Shanshan Luan, Suyu Gao, Li Cheng, Bin Hao, Jiacheng Li, Yao Chen, Xuemei Hou, Lixia Chen, Hua Li

**Affiliations:** ^1^ School of Traditional Chinese Materia Medica, Wuya College of Innovation, Key Laboratory of Structure-Based Drug Design & Discovery, Ministry of Education, Shenyang Pharmaceutical University, Shenyang 110016, China; ^2^ Hubei Key Laboratory of Natural Medicinal Chemistry and Resource Evaluation, School of Pharmacy, Tongji Medical College, Huazhong University of Science and Technology, Wuhan 430030, China; ^3^ Livzon Pharmaceutical Group Inc., Zhuhai 519090, China

**Keywords:** Ilaprazole, PPI, cancer, drug repurposing, TOPK inhibitor

## Abstract

T-cell-originated protein kinase (TOPK) is highly and frequently expressed in various cancer tissues and plays an indispensable role in the mitosis of cancer cells, and therefore, it is an important target for drug treatment of tumor. Ilaprazole was identified to be a potent TOPK inhibitor. The data indicated that ilaprazole inhibited TOPK activities with high affinity and selectivity. *In vitro* studies showed that ilaprazole inhibited TOPK activities in HCT116, ES-2, A549, SW1990 cancer cells. Moreover, knockdown of TOPK in these cells decreased their sensitivities to ilaprazole. Results of an *in vivo* study demonstrated that gavage of ilaprazole in HCT116 colon tumor-bearing mice effectively suppressed cancer growth. The TOPK downstream signaling molecule phospho-histone H3 in tumor tissues was also decreased after ilaprazole treatment. Our results suggested that ilaprazole inhibited the cancer growth by targeting TOPK both *in vitro* and *in vivo*.

## INTRODUCTION

TOPK (T-lymphokine-activated killer cell-originated protein kinase) is a Ser/Thr protein kinase, also known as PBK or PDZ-binding kinase, which belongs to the MEK protein family [1, 2]. The PBK/TOPK protein is up-regulated in certain types of cancer cells such as breast, colon, lung and glioma cancer cells [3–5], but is difficult to be detected in normal tissues except several fetal tissues and germ cells of the testis [6]. In molecular studies, over-expression of TOPK involves in mitotic checkpoint of tumor cells, cell apoptosis and inflammation [7]. In addition, TOPK was also reported to increase cell migration by modulating a PI3K/PTEN/AKT-dependent signaling pathway [8]. Furthermore, histone H3 could be phosphorylated at Ser10 by TOPK both *in vivo* and *in vitro*, which could mediate cell growth-promoting effects [9]. Based on these findings, TOPK has become a novel target for cancer treatment attracting more and more attentions [10]. Some TOPK inhibitors, such as HI-TOPK-032 [11] and OTS964 [12] have been reported in 2012 and 2014, respectively. However, none of them had been in clinical trials because of their toxicities or poor pharmacokinetic properties.

Proton pump inhibitors (PPIs), like omeprazole and esomeprazole, are standard treatments for gastric acid-related diseases, acting as potent inhibitors of the gastric acid pump [13]. A few studies showed that PPIs could enhance antitumor effects of cytotoxic drugs in drug-resistant tumors by inhibiting V-ATPase activities or affecting homeostasis, especially acidic vesicles trafficking in cancer cells [14–16]. In our previous study, pantoprazole (PPZ), a PPI was identified as a TOPK inhibitor which could suppress growth of colorectal cancer cells [17]. This is the first report that PPIs can directly inhibit cancer growth via a proton pump independent mechanism.

Inspired by our results of pantoprazole, we speculated that other PPIs may also be developed as TOPK inhibitors. Based on the availability, another six proton pump inhibitors (PPIs) in clinical use were screened against TOPK by virtual ligand screening method, microscale thermophoresis (MST) and cytotoxic activity assay. Ilaprazole, a novel proton pump inhibitor, was found to show potent antitumor activities through the inhibition of TOPK-specific kinase function.

## RESULTS

### Identification of ilaprazole as TOPK inhibitors

**Figure 1:**
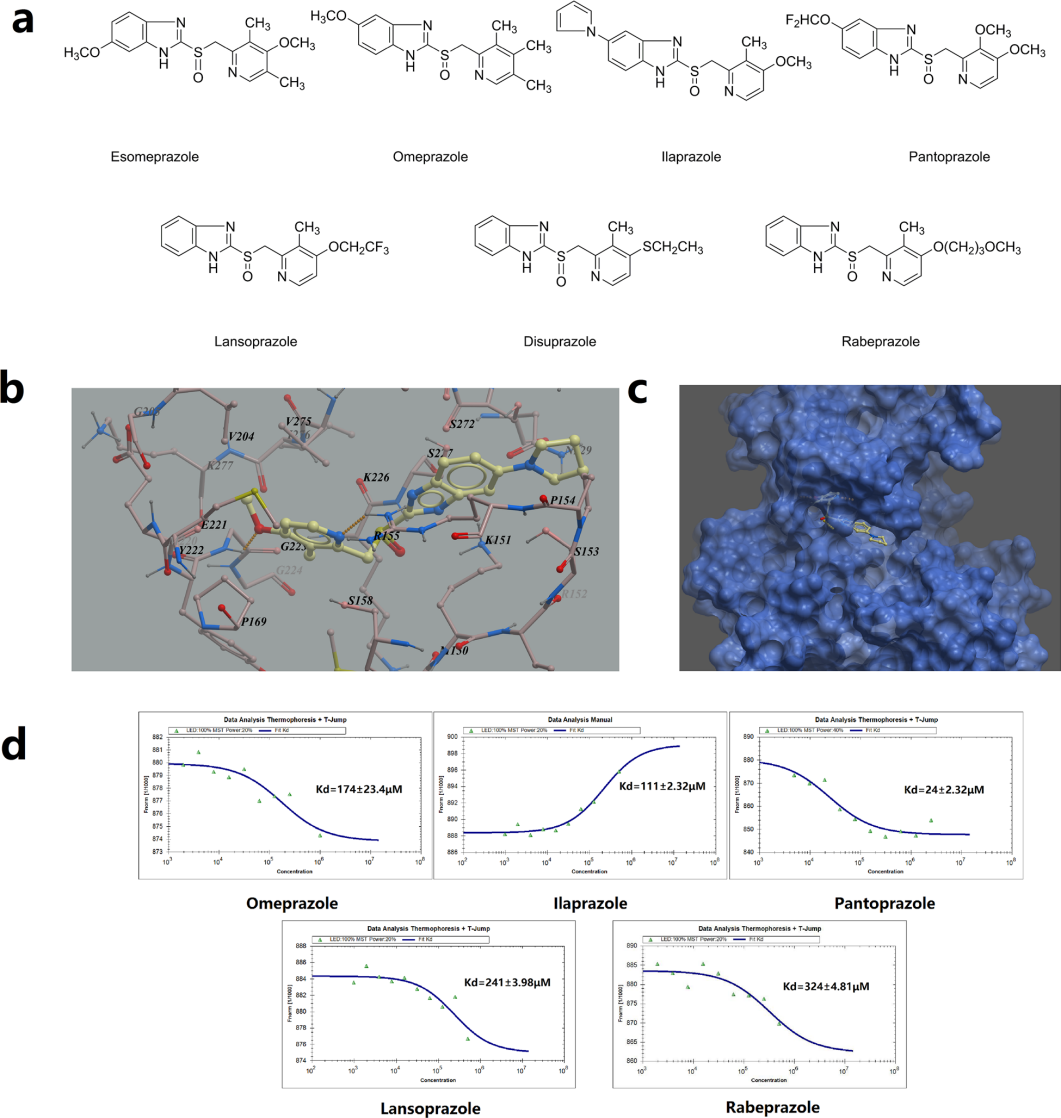
Virtual ligand screening and MST assay identifies the binding of ilaprazole to TOPK **(a)** Chemical structures of PPIs. **(b)** Detailed view of ilaprazole binding in the ligand binding pocket. **(c)** Overlay of one monomer of the TOPK–ilaprazole binary complex. **(d)** Measurement of affinity between screened drugs with TOPK by MST, and the resulting binding curves were shown.

In our previous study, we identified proton pump inhibitor pantoprazole as a potent TOPK inhibitor [13]. It suppressed cancer cell growth both *in vitro* and *in vivo*. Encouraged by this finding, we then sought to determine if any drugs from the same class could also potentially inhibit TOPK activities. The homology model of human TOPK was constructed by using the X-ray structure of the mixed-lineage kinase MLK1 (PDB code: 3DTC) as the template, which has 29% of sequence identities to the human TOPK. The docking was performed by using ICM 3.8.1 modeling software (MolSoft LLC, San Diego, CA). As is shown in the Figure 1a, all of the PPIs compounds are structural analogues, weakly basic 2-pyridylmethyl-sulfinyl-benzimidazoles derivatives. The virtual screening results of seven prazoles were shown in Table 1. The top three compounds with highest ICM scores were ilaprazole, lansoprazole and omeprazole. From the generated docking model of ilaprazole, hydrogen bonds were predicted between the methoxyl oxygen of ilaprazole with Gly223, pyridine nitrogen with Arg155, respectively. Also, π-π stacking interactions may form between the pyridine ring of ilaprazole with the pyrrole ring of Pro169, the pyrrole ring of ilaprazole with the pyrrole ring of Pro154 (Figure 1b, 1c).

**Table 1:** Predicted binding free energies and inhibitory activities of screening hits

Prazoles	ICM scores ^a^	Kd (μM) ^b^	IC_50_ for HCT116 cell activities (μM)
Esomeprazole	−26.24	n.b^c^	>500
Omeprazole	−26.24	174.3 ± 3.1	348.3 ± 7.3
Ilaprazole	−31.88	111.0 ± 2.3	40.0 ± 0.6
Pantoprazole	−25.65	24.2 ± 2.3	153.0 ± 8.5
Lansoprazole	−27.35	241.6 ± 5.2	102.9 ± 4.1
Disuprazole	−19.36	n.b^c^	>500
Rabeprazole	−8.63	324.2 ± 6.3	>500

a. Docking score/interaction potential of compounds with TOPK (kcal/mol).

b. The Kd value is automatic calculated by the curve fitting, and presents as means ± SD for three experiments.

c. n.b. is no clear binding detected in the MST measurement.

To validate results of the virtual ligand screening, we employed microscale therm ophoresis method (MST) to assay the binding affinity between compounds and TOPK. This technology detected fluorescent changes of molecules during thermophoresis, to quantify protein-protein interactions or protein-small molecule interactions with high sensitivity and low sample cost. Among PPIs assayed, except pantoprazole, ilaprazole exhibited the lowest equilibrium dissociation constant (Kd) of 111.0 ± 2.3 μM (Table 1, Figure 1d), which meant the strongest binding.

Attempts were then made to determine whether PPIs could affect growth of colon cancer cells. As shown in Table 1, seven PPIs exhibited certain levels of inhibition for TOPK over-expressed HCT116 cells with IC_50_ values from 43.0 to 348.3 μM. The activity ranking was ilaprazole > lansoprazole > pantoprazole for the best three prazoles. Ilaprazole, the one with strongest inhibitory effects, was chosen for further investigations.

### TOPK expression in cancer cell lines

**Figure 2:**
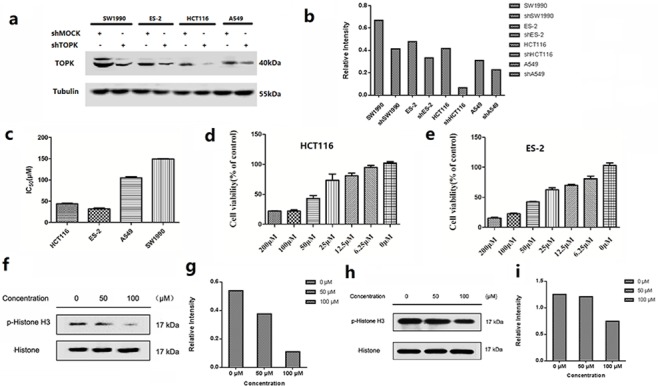
*In vitro* antiproliferative activity of ilaprazole **(a, b)** Expression of TOPK in lung cancer (A549), colon cancer (HCT-116), human ovarian cancer (ES-2) and pancreatic cancer cell (SW1990) lines. Tubulin was examined to serve as a loading control. **(c)** Graph indicates IC_50_ values of ilaprazole in 4 TOPK-positive cancer cell lines. **(d, e)** The cell proliferation rate of HCT116 and ES-2 were remarkably decreased by ilaprazole. **(f, g)** Ilaprazole inhibits TOPK activity in HCT116 cells. The expression level of phosphorylated histone H3 (Ser10) and histone H3 was detected by western blot analysis. **(h, i)** Ilaprazole inhibits TOPK activity in ES-2 cells. The expression level of phosphorylated histone H3 (Ser10) and histone H3 was detected by western blot analysis. The relative intensity of p-Histone and Histone was calculated by an image J software.

TOPK protein levels were measured by western blot in cancer cell lines derived from various cancer types, such as lung cancer (A549), colon cancer (HCT116), human ovarian cancer (ES-2) and pancreatic cancer cell (SW1990). High expression levels of TOPK were observed in all these cell lines (Figure 2a, 2b). The growth inhibitory effects of ilaprazole on each cell line were measured, and obtained IC_50_ values were ranging from 30.10 to 148.21 μM (Figure 2c). Ilaprazole exhibited potent inhibitory activities against the growth of HCT116 cells (IC_50_ = 40.0 ± 0.6 μM) and ES-2 cells (IC_50_ = 33.2 ± 1.0 μM) (Figure 2d, 2e).

### Ilaprazole suppresses TOPK activities *in vitro* and induces apoptosis

**Figure 3:**
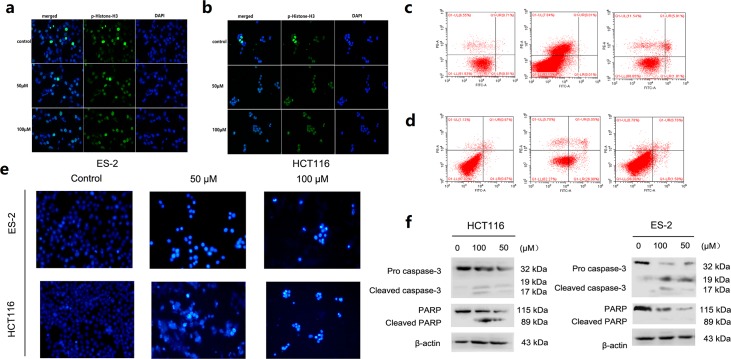
Suppression of TOPK activities and induces apoptosis after treatment with Ilaprazole **(a, b)** Immunocytochemical staining analysis of p-Histone H3 in ES-2 and HCT116 cells. **(c, d)** ES-2 and HCT116 cells were treated for 24 h with 50 or 100μM of ilaprazole and then processed for FACS by using Annexin V/propidium iodide staining. **(e)** The cells were treated with ilprazole (100 and 50 μM) for 24 h, then stained with Hoechst 33342 and observed through a fluorescence microscope (×200 magnification). Bar = 20 μm. **(f)** The cells were incubated with increasing concentrations (0, 50, 100 μM) of ilprazole for 24 h, followed by western blot analysis for the detection of PARP and caspase-3 levels. Data are represented as mean ± standard deviation from triplicate experiments.

TOPK proteins were expressed mainly around chromosomal surfaces in mitotic cells, particularly at prophase and metaphase, so the histone was chosen as the substrate for TOPK activity evaluation. Some reports confirmed that TOPK could phosphorylate histone H3 *in vitro* and *in vivo*. Hence, we examined whether ilaprazole could suppress TOPK activities in HCT116 and ES-2 cells by western blot analysis. Results showed that the phosphorylation of histone H3 (Ser10) was substantially attenuated in a dose-dependent manner after treatment with ilaprazole (Figure 2f–2i). These results suggested that TOPK was a direct target for ilaprazole to suppress cancer cell growth. Immunocytochemical staining results showed that TOPK-mediated p-Histone H3 was substantially decreased dose-dependently with ilaprazole treatment. (Figure 3a, 3b, Supplementary Figure 1a, 1b)

Effects of ilaprazole on the cellular apoptosis of HCT116 and ES-2 cells were further investigated. Cells were pre-treated with different concentrations of ilaprzole for 24 h, and then the cellular apoptosis was measured by using flow cytometry. Normalization to the DMSO-treated controls revealed that ilaprazole at 50 μM induced 32.03% (HCT116) and 7.62% (ES-2) apoptotic death compared with 2% of the control (Figure 3c, 3d).

In order to determine effects of cell apoptosis induced by ilaprazole, the cells treated with 100 and 50 μM ilaprazole were stained with Hoechst 33342 and the typical morphological features of later stage apoptosis increased condensation of chromatin material and fragmentation of the nuclei were observed (Figure 3e).

Ilaprazole was also found to induce the cleavage of poly-(ADP-ribose) polymerase (PARP), a DNA repair regulatory protein. Furthermore, the active cleavage forms of caspase-3 were observed after treating with ilaprazole for 24 hours, indicating that it might induce apoptotic cell death by activation of caspases in a dose-dependent manner (Figure 3f). These results suggested that ilaprazole might induce apoptosis in HCT116 and ES-2 cells. In addition, the cell cycle distributions of HCT116 cells were examined after treating with increasing doses of ilaprazole for 48 hours. Flow cytometric analysis demonstrated that ilaprazole induced cell cycle arrested at the G1 phase in HCT116 cells, as shown in Supplementary Figure 2.

### The inhibition of TOPK by ilaprazole is dependent on the abundance of TOPK

To investigate the growth-promoting role of TOPK in cancer cells, the expression of endogenous TOPK was knocked down in four cancer cells. By lentiviral infection, HCT116, ES-2, A549 and SW1990 cell lines stably expressing low levels of TOPK (shTOPK) were established. As the control, cells were infected with shMock. The abundance of TOPK in these cells was confirmed by western blot analysis (Figure 2a).

**Figure 4:**
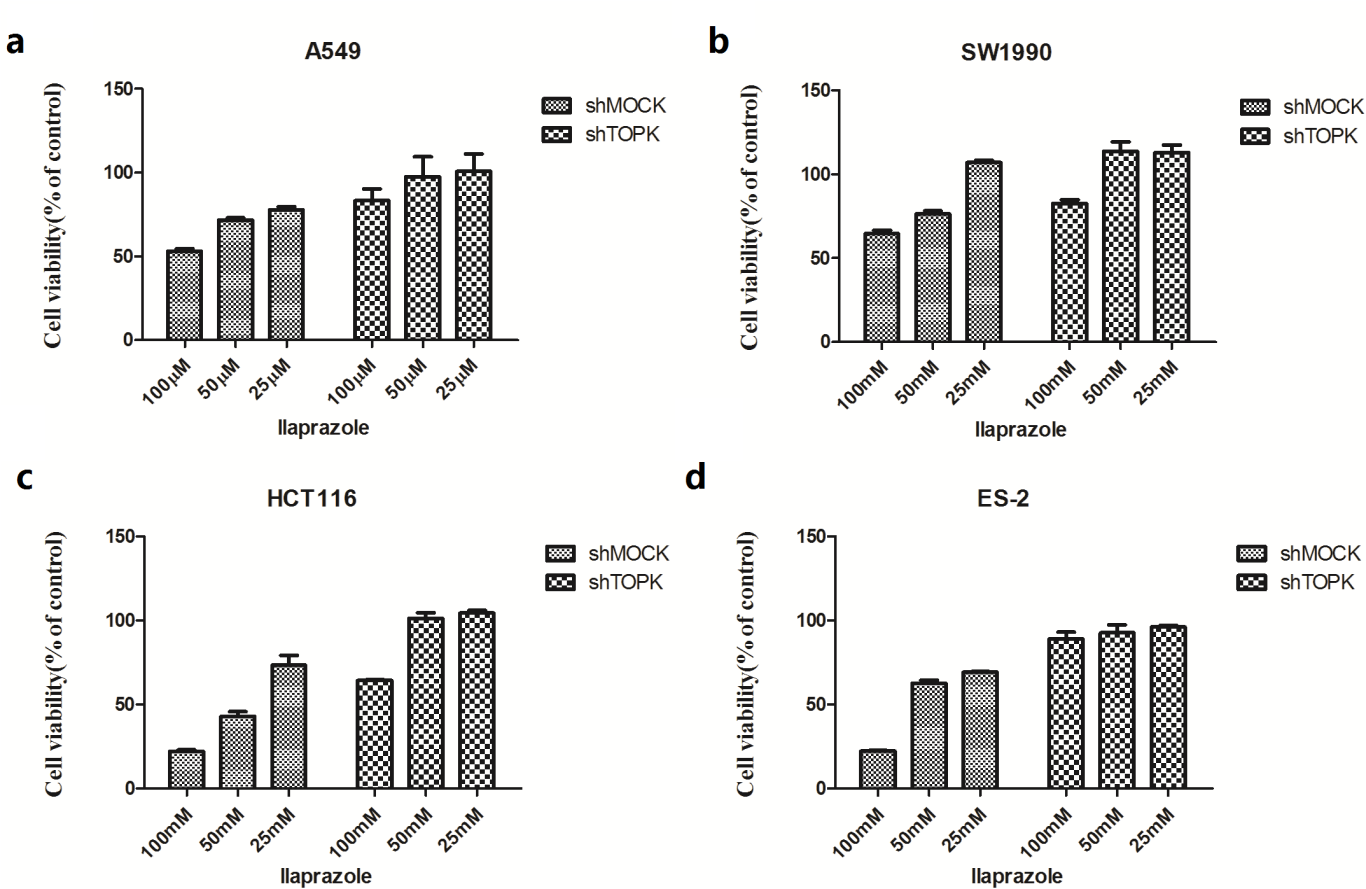
The inhibition of TOPK by ilaprazole is dependent on the abundance of TOPK Ilaprazole has less effect on anchorage-independent cell growth of shTOPK transfected cells than that of shMOCK cells. The effect of ilaprazolewas examined in shMock and knockdown cell lines with shTOPK. Cells were incubated for 48 hours and growth was determined by cck8 assay.

Effects of ilaprazole on the growth of shMock and shTOPK infected colon cancer cells were assessed by CCK8 assay. Results indicated that cells infected with shTOPK were resistant to the inhibitory effect of ilaprazole on the growth compared with cells infected with shMock (Figure 4). These findings suggested that anticancer activities induced by ilaprazole were dependent on the TOPK expression.

### Ilaprazole suppresses tumor growth by inhibiting TOPK activity *in vivo*

**Figure 5:**
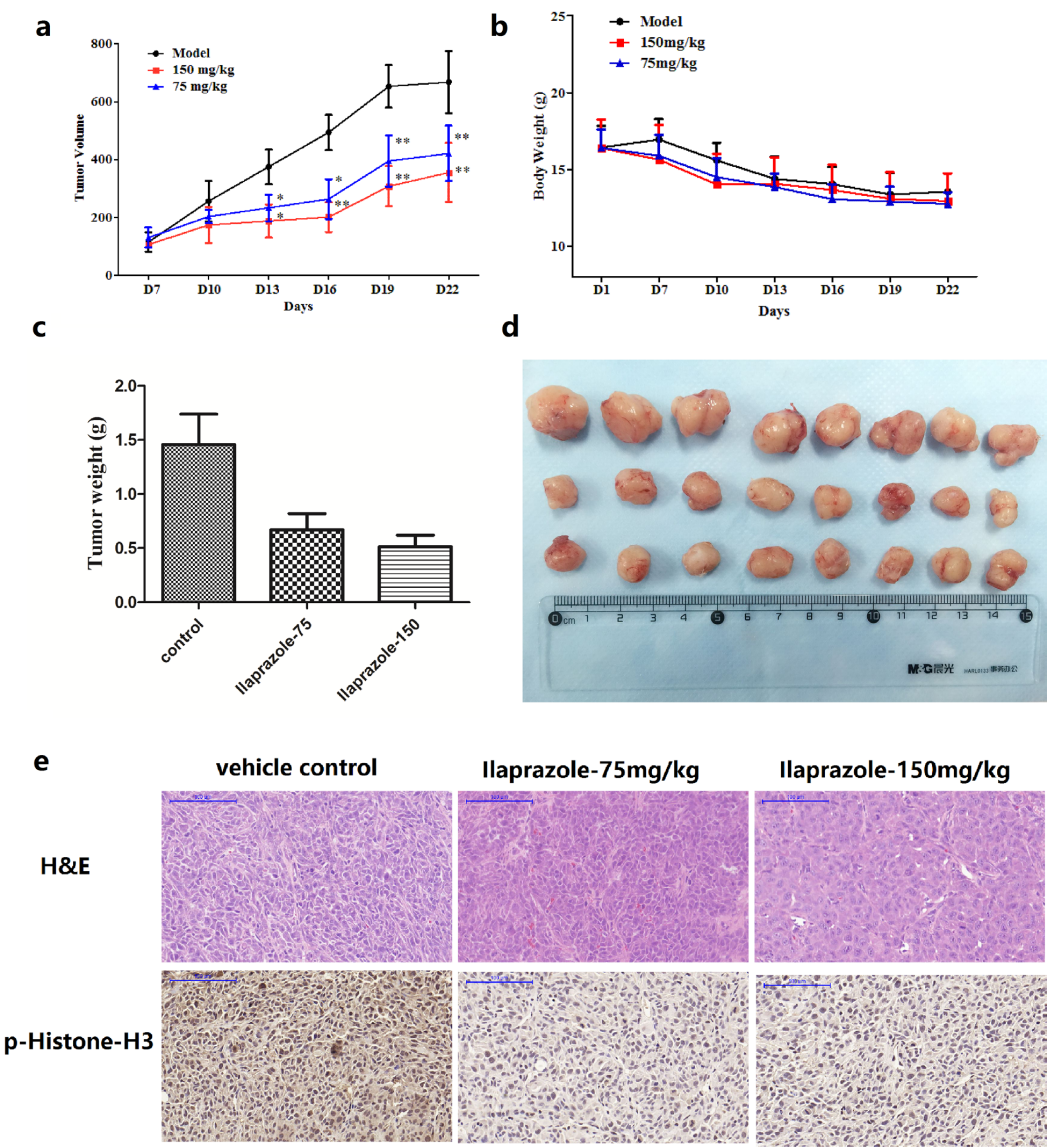
Effect of ilaprazole on colon cancer growth in an HCT116 xenograft mouse model **(a)** The average tumor volume of vehicle-treated control mice (n = 8) and ilaprazole treated mice (n = 8, 75 or 150 mg/kg per day via gavage) plotted over 21 days after tumor cell injection. **(b)** Ilaprazole has no effect on mouse body weight. **(c, d)** After 2 weeks treatment, differences in tumor weight and size are shown. The asterisk * indicates a significant increased tumor size (P < 0.05) in the vehicle-treated group compared with the ilaprazole-treated group as determined by one-way analysis of variance. **(e)** Ilaprazole inhibits expression of phosphorylated histone H3 *in vivo*. Immunohistochemistry analysis was used to determine the level of phosphorylated histone H3 in tumor tissues.

After injected with HCT 116 cells, CB-17/Icr-scid mice were divided into three matched groups including a vehicle control and two ilaprazole treating groups (75, 150 mg/kg). After establishing palpable tumors, mice were administrated with ilaprazole or vehicle via gavage daily. Estimated tumor volumes of both treating groups were less than that of the control group throughout the treatment. Significant decreases of the tumor volume were observed after 2 weeks' treatment (Figure 5a, Figure 5d). Animals were then scarified and the average tumor weights were measured and calculated. The tumor weight of the control group was 1.45 ± 0.33 g, whereas the tumor weights of low-dose and high-dose treated groups were 0.67 ± 0.21g and 0.52 ± 0.11g, respectively (Figure 5c), representing significant reduction. Moreover, no obvious hepatotoxicity and nephrotoxicity, or differences of average body weights among the three groups were observed throughout the study (Figure 5b, Supplementary Figure 3), which revealed that the dosage of ilaprazole used in this experiment had no obvious side effects on animals' growth. To further determine whether antitumor effects of ilaprazole were associated with its inhibition of TOPK activities, tumor extracted from each group were prepared and analyzed for the phosphorylation of histone H3 by immunohistochemistry analysis. Results showed that expression levels of phosphorylated histone H3 were substantially decreased in ilaprazole-treated groups compared with the vehicle group (Figure 5e). Overall, the results indicated that ilaprazole could suppress tumor growth by inhibiting TOPK activities *in vivo*.

## DISCUSSION

Previous studies have shown that TOPK, a novel serine-threonine kinase, belongs to the MAPKK family. Many groups have reported that TOPK was associated with oncogenic cellular functions including tumor development, cancer growth, and anti-apoptotic effects [1–2, 7–9]. These findings suggest that TOPK is a novel target for development of anticancer agents.

Based on our previous finding that pantoprazole was the inhibitor of TOPK, we screened another 6 prazoles for potential TOPK inhibitory activities, and identified ilaprazole as another potent TOPK inhibitor. According to our results, PPIs' inhibitory effects on TOPK were affected by the presence of steric hindrance near pyridine group and substituents on the benzimidazole. Apparently, disuprazole and rabeprazole exhibited lower ICM scores, presumably because of the larger substituents ethylthio group and methoxypropoxy group respectively, which revealed that the substituent at 4-position of pyridine ring influenced the inhibitory potential as a result of steric hindrance. Results of MST assay and activities on HCT116 cells also supported this hypothesis that steric hindrance of substituents of pyridine ring was one of the crucial factors affecting inhibitory activities. Another important factor affecting inhibitory activities was the substituent at 5-position of the benzimidazole. As an electron-donating group, methoxyl group at 5-position may promote the ability to inhibit TOPK. Consequently, pantoprazole and omeprazole showed a stronger affinity with TOPK. By forming π-π stacking interaction with the pyrrole ring of Pro154, pyrrole ring at 5-position also promoted the ability to inhibit TOPK, consequently making ilaprazole a promising inhibitor against TOPK with lower IC_50_ value. When physicochemical and pharmacokinetic properties are taken into consideration, it is not difficult to illuminate that ilaprazole exhibited the strongest inhibitory effects *in vitro* and *in vivo*. The logP value of ilaprazole is larger than that of pantoprazole according to the data from SciFinder Academic database (logP value of ilaprazole: 2.882 ± 1.045; logP value of pantoprazole: 1.571 ± 1.065). This may offer parts of the explanation for the reason why ilaprazole showed stronger inhibitory effects *in vitro* and *in vivo*, while its binding affinity with TOPK was weaker than pantoprazole.

Use of ilaprazole has been shown to inhibit the growth of cancer cells both *in vitro* and *in vivo*. Our results indicated that TOPK highly expressed cancer cells were strongly inhibited by ilaprazole and the anticancer activity induced by ilaprazole was dependent on the TOPK expression. The phosphorylations of histone H3 (Ser10), the downstream substrate of TOPK, were strongly reduced in cancer cells by treating with ilaprazole. Notably, the phosphorylations of histone H3 (Ser10) were significantly inhibited in ilaprazole-treated tumor tissues.

As we know, long-term and high-dose PPIs treatment has been shown to be well tolerated in patients with few side effects. [18–20] Therefore, repositioning PPIs as anticancer drugs will be unlikely to have added toxicities. Although PPIs has been proposed as the adjuvant drugs for cancer treatment, and even a clinical trial was performed to evaluate the use of pantoprazole in combination with doxorubicin for advanced cancer patients with solid tumors [21, 22]. However, whether PPIs directly suppress tumor growth remained to be determined.

Ilaprazole is a new PPI that was developed by Livon Pharmaceutical Group, approved in China for the treatment of duodenal ulcer in 2007. The toxicological data showed that the LD_50_ of ilaprazole was more than 5000 mg/kg in rats [23]. In this study, we have demonstrated that oral administration of ilaprazole 75 mg/kg and 150 mg/kg had no obvious hepatotoxicity and nephrotoxicity, or difference of average body weights. Considering the low toxicities of ilaprazole and effective anti-tumor activities observed in this study, it would be suitable to expand its applications to targeted cancer therapy.

In conclusion, we discovered ilaprazole as a novel and specific TOPK inhibitor both *in vitro* and *in vivo*. These findings should be useful for further development of drugs targeting TOPK by employing ilaprazole as the lead compound. Future studies will focus on the efficacy of ilaprazole and characterize its therapeutic potential against other cancers with high expression levels of TOPK.

## MATERIALS AND METHODS

### Reagents and antibodies

Antibodies against phospho-Histone H3, TOPK, Histone H3, PAPR, caspase 3 were obtained from “Cell Signaling Technology” (USA), *β*-actin and horseradish peroxidase (HRP) conjugated secondary antibody from rabbit and mouse were purchased from “Santa Cruz” (USA) and “Protein Tech Group” (USA), respectively. The chemiluminescence's detection kit ECL Plus was from “GE Healthcare” (USA). The Dulbecco's Modified Eagle medium (DMEM), Roswell Park Memorial Institute medium (RPMI 1640), phosphate buffered saline (PBS), trypsin, fetal bovine serum (FBS) were purchased from Thermo Scientific. Hoechst 33342 was purchased from Sigma-Aldrich (St. Louis, MO, USA). All other common chemicals, solvents and reagents were of highest grade available from various commercial sources.

3-(4,5-Dimethylthiazol-2-yl)-2,5-diphenyltetrazolium bromide (MTT), MDC, Rhodamine 123, and Hoechst 33258 were purchased from Sigma-Aldrich (St. Louis, MO, USA). Antibodies against PARP, Bcl-xL, Bax, cytochrome c, caspase-8, caspase-9, β-actin, p62, LC3-II, Beclin-1, p-p38, p-38, p-JNK, JNK, p-Erk, Erk, Histone H3 and NF-κB were purchased from Cell Signaling Technology (CST, Beverly, MA). NF-κB SN50 was from Merck Millipore (USA).

### Cell culture

Human cancer cells lines, HCT116 (ATCC # CCL-247™), ES-2 (ATCC # CRL-1978™), A549 (ATCC # CRM-CCL-185™) and SW1990 (ATCC # CRL-2172™), were cultured under the recommendations of their respective depositors. All cell lines were authenticated by STR analysis from Shanghai Zhong Qiao Xin Zhou Biotechnology Co.,Ltd.

The cells were grown for 2-3 days, after reaching 90% of confluence, were then harvested by exposure to 0.25% Trypsin-EDTA solution and then passed into new T-25 tissue culture flasks. The cell cultures were performed following the instructions of ATCC.

### Protein expression, purification

Human TOPK gene (Accssion number: NM_018492) was cloned into the pET26b vector (Novagen). The expression construct pET26b-TOPK was transformed into *Escherichia coli* strain BL21 (DE) (Invitrogen) and selected on kanamycin plates. The transformed cells were cultivated in Luria-Bertani (LB) media at 37°C in the presence of kanamycin until the optical density (OD) reached 0.8. Cells were then induced with 0.4 mM IPTG (isopropyl-*β*-*D*-1-thiogalactopyranoside) for 16 h at 20°C. The cells harvested by centrifugation were lysed by ultrasonication on ice in a buffer containing 20 mM Tris, pH 8.5, 200 mM NaCl, 5 mM mercaptoethanol, 0.1% TritonX-100, and 5% glycerol.

Soluble hexa-histidine tagged TOPK was bound to Ni-agarose affinity resin (Qiagen), washed with buffer A (20 mM Tris, pH 8.5, 200mM NaCl, and 10 mM imidazole), and eluted with buffer B (20 mM Tris, pH 8.8, 250 mM NaCl, and 150 mM imidazole).

### Microscale thermophoresis (MST)

Recombinant TOPK was labeled with the Monolith NT™ Protein Labeling Kit RED (Cat # L001) according to the supplied labeling protocol. Labeled TOPK was used in a concentration of 50 nM. The samples were diluted in a 20 mM HEPES (pH 7.4) and 0.5 (v/v) % Tween-20. The prazoles stocks were dissolved in 10% DMSO in a concentration of 1 mM. 1mM prazoles were used as the highest concentration for serial dilutions. After 10minutes incubation at room temperature, the samples were loaded into Monolith^TM^ standard-treated capillaries and the thermophoresis was measured at 25°C after 30 minutes incubation on a Monolith NT.115 instrument (NanoTemper Technologies, München, Germany). Laser power was set to 20% using 30 seconds on-time. The LED power was set to 100%. The dissociation constant Kd values were fitted by using the NTAnalysis software (NanoTemper Technologies, München, Germany) [24].

### Homology modeling and molecular docking

With the crystal structure of the mixed-lineage kinase MLK1 (PDB code: 3DTC) as the template, a homology model of human TOPK (accession number: NP_060962) was constructed by using MODELLER (an automated homology modeling program) [25, 26]. Chemical structures of prazoles were input as mol2 files for docking [21]. The docking was performed by using ICM 3.8.1 modeling software on an Intel i7 4960 processor (MolSoft LLC, San Diego, CA) [27]. Ligand binding pocket residues were selected by using graphical tools in the ICM software, to create the boundaries of the docking search. In the docking calculation, potential energy maps of the receptor were calculated using default parameters. The compounds were imported into ICM and an index file was created. The conformational sampling was based on the Monte Carlo procedure, and finally the lowest-energy and the most favorable orientation of the ligand was selected.

### Cell viability assay

To estimate cell viability, human colon cancer HCT 116, ES-2, A549 and SW1990 cells (5000 cells/well) were seeded in 96-well plates for 24 h at 37°C in a 5% CO_2_ incubator. The attached cells were fed with fresh medium containing various concentrations of ilaprazole (0-100 μM) for additional 24 h and 48 h. After culturing for various times, the cytotoxicity of ilaprazole was measured using a cck8 assay kit according to the manufacturer's instructions. All experiments were performed in triplicate, and the mean absorbance values were calculated. The results are expressed as the percentage of inhibition that produced a reduction in absorbance by ilaprazole treatment compared with the non-treated cells (control).

### Isolation of histone H3

Histones were extracted from cells by disrupting the cells with NETN buffer [150 mM NaCl, 1 mM EDTA, 20 mM Tris-HCl (pH 8.0), 0.5% nonionic detergent IGEPAL CA 630(NP-40), Sigma]. The insoluble fraction was pelleted for 5 minutes in a microcentrifuge (8,400 rpm). Nuclei were extracted with 0.1 M HCl to isolate the total histones. The samples were precipitated with 1 M Tris- HCl (pH 8.0) and then resuspended in ddH2O.

### Western blot analysis

The harvested cells were lysed with lysis buffer (50 mM Tris-HCl (pH 7.4), 150 mM NaCl, 1 mM EDTA, 1 mM EGTA, 10 mg/mL aprotinin, 10 mg/mL leupeptin, 5 mM phenylmethanesulfonyluoride (PMSF), 1 mM dithiolthreitol (DTT) containing 1% Triton X-100). Insoluble debris was removed by centrifugation at 12000 rpm for 15 minutes, and protein's content was determined using Bradford reagent (Bio-Rad, USA). Lysate protein (20-40 μg) was subjected to 10% SDS-PAGE and electrophoretically transferred to polyvinylidene difluoride membranes (PVDF) (Millipore, USA). The membranes were blocked with 5% non-fat milk or 5% BSA for 1 hour and then incubated with the specific primary antibodies respectively at 4°C overnight. Protein bands were visualized using an enhanced chemiluminescence reagent (ECL Plus) (GE Healthcare, USA) after hybridization with a HRP conjugated secondary antibody. Band density was quantified using the Image J software program (NIH).

### Apoptosis analysis

Apoptotic cells were assessed by staining PBS washed cells with phycoerythrin (PE)-conjugated annexin-V (BD Pharmingen) for 10-15 minutes in incubation buffer (Annexin-V-FLUOS Staining Kit, Roche). Fluorescence was recorded by flow cytometry using the FACS Aria (BD Bio sciences).

### Hoechst 33342 staining

After incubation with ilaprazole for 24 h, the cells were stained with Hoechst 33342 at 37°C for 30 min, and then the morphology was observed by fluorescence microscopy (Zeiss, OBSERVER D1/AX10 cam HRC).

### Lentiviral infection

The lentiviral expression vectors, including Gipz-shTOPK, and packaging vectors, including pMD2.0G and psPAX, were purchased from Addgene Inc. To prepare TOPK viral particles, each viral vector and packaging vectors (pMD2.0G and psPAX) were transfected into HEK293T cells using jetPEI following the manufacturer's suggested protocols. “The target sequences of oligo siRNAs were as follows: 5′-GTGTGGCTTGCGTAAATAA -3′ for TOPK to produce lentiviral particles.”

The transfection medium was changed at 4 hours after transfection and then cells were cultured for 36 hours. The viral particles were harvested by filtration using a 0.45-mm syringe filter, then combined with 8 mg/mL of polybrene (Millipore) and infected into 60% confluent HCT-116 cells overnight. The cell culture medium was replaced with fresh complete growth medium for 24 hours and then cells were selected with puromycin (1.5 mg/mL) for 36 hours. The selected cells were used for experiments [28, 29].

### *In vivo* xenograft mouse model

CB-17/Icr-scid mice were purchased from Beijing HFK Bioscience CO., LTD (Beijing, China). The animals were maintained under ‘specific pathogen free’ conditions. The mice were randomly divided into three groups: (i) vehicle group (n = 8); (ii) 150 mg/kg ilaprazole-treated group (n = 8); (III) 75 mg/kg ilaprazole-treated group. HCT 116 cells were inoculated subcutaneously (2 × 10^6^ cells) into the left flank of each mouse in all groups. Treatment was started after 4 days of cell injection. The duration of the animal study was 19 days. The tumor volume was calculated from measurements of 3 diameters of the individual tumor based on the following formula:

tumor volume (mm^3^) = (length × width × height × 0.52). The mice were monitored until tumors reached 1 cm^3^ total volume, at which the mice were euthanized and the tumors were extracted. The tumors were dissected and sent for immune-histochemical analysis.

### Statistical analysis

Statistical analysis of the data was performed using Graph Pad Prism 5.0 software. The data were expressed as the means ± SD. Values were analyzed using SPSS version 12.0 software by one-way analysis of variance (ANOVA), and *p* < 0.05 was considered statistically significant.

## SUPPLEMENTARY MATERIALS FIGURES



## Abbreviations

PPIs: Proton Pump Inhibitors
TOPK: T-cell-originated protein kinase
PPZ: pantoprazole
MST: microscale thermophoresis
CCK8: Cell Counting Kit-8
IPTG: isopropyl *β*-*D*-1-thiogalactopyranoside
PMSF: phenylmethanesulfonyluoride
PVDF: Polyvinylidene
H&E: Hematoxylin-eosin
