# Supplementary material for: Proton pump inhibitor ilaprazole suppresses cancer growth by targeting T-cell-originated protein kinase
Source: Oncotarget. 2017 Mar 27;8(24):39143–53. doi: 10.18632/oncotarget.16609 (PMC5503601; doi:10.18632/oncotarget.16609)
Supplement: Supplementary file 1 [file oncotarget-08-39143-s001.pdf]

Proton pump inhibitor ilaprazole suppresses cancer growth by targeting T-cell-originated protein kinase

SUPPLEMENTARY FIGURES

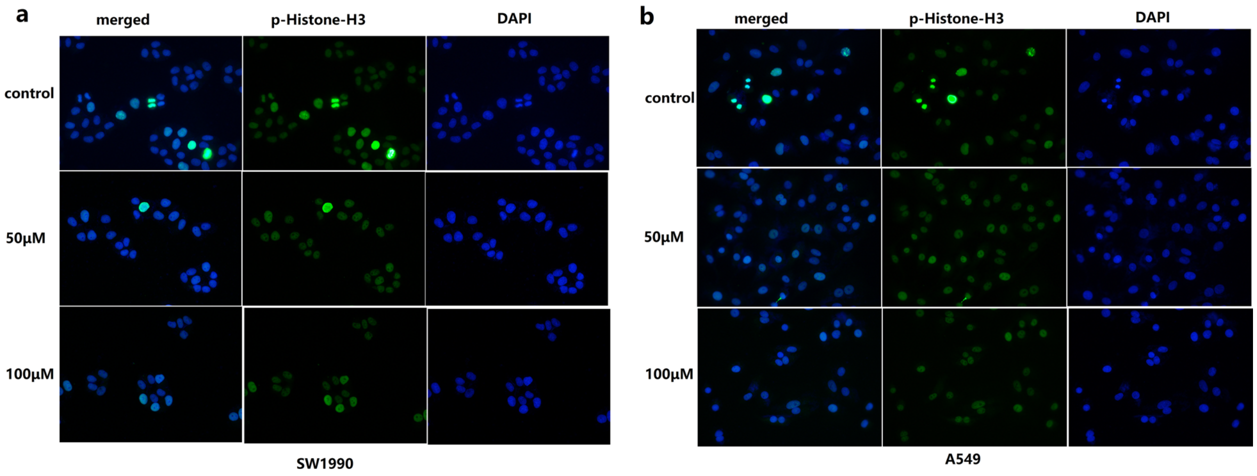

Supplementary Figure 1: Immunocytochemical staining analysis of p-Histone H3 in SW1990 and A549 cells.

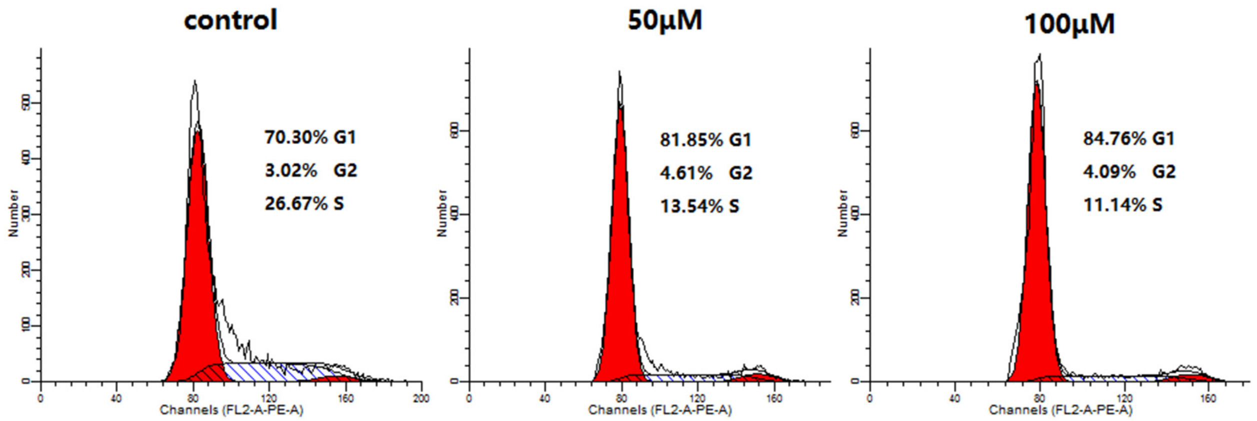

Supplementary Figure 2: Ilaprazole induced cell cycle arrested at the G1 phase in HCT116 cells.

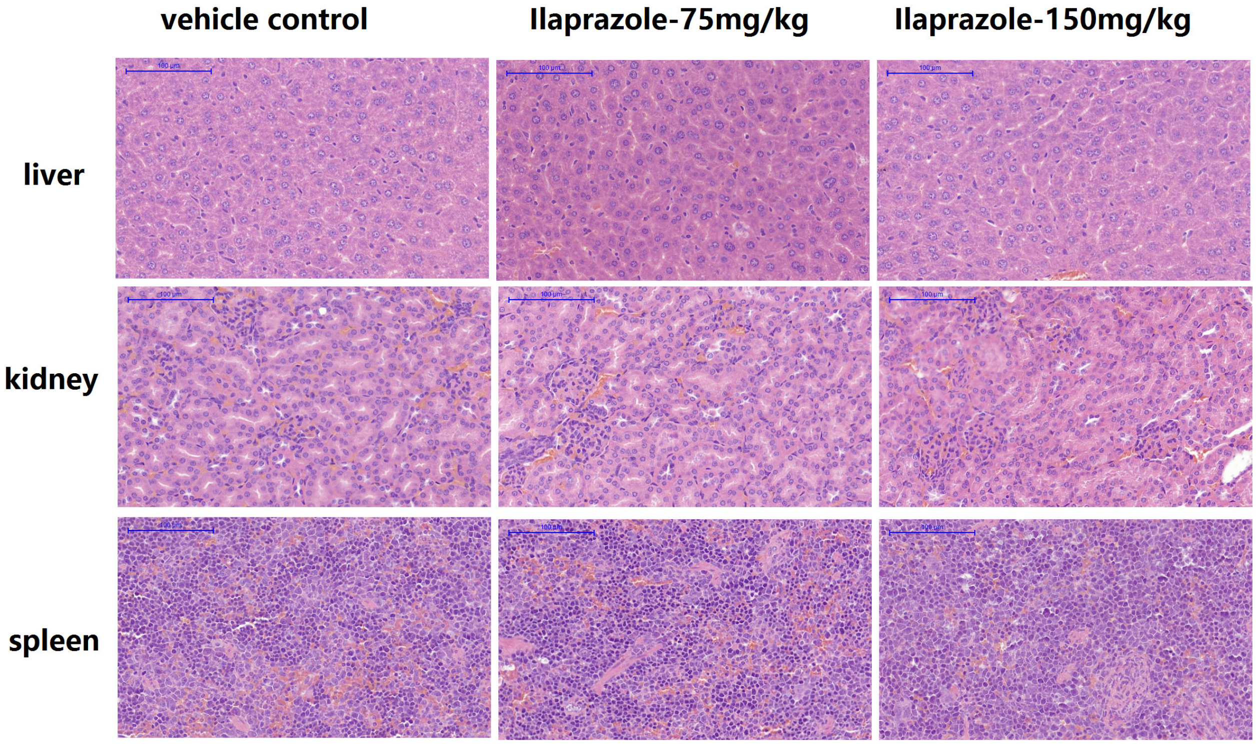

Supplementary Figure 3: Microstructures of sections of the liver, kidney and spleen in control mice and mice treated with doses of ilaprazole daily for 15 days. No significant damage was observed in all treatment groups. Magnification 400×(DXIT 1200, Nikon, Japan).
